# Immunoinformatic evaluation for the development of a potent multi-epitope vaccine against bacterial vaginosis caused by *Gardnerella vaginalis*

**DOI:** 10.1371/journal.pone.0316699

**Published:** 2025-02-27

**Authors:** Hamid Motamedi, Saeed Shoja, Maryam Abbasi

**Affiliations:** 1 Asadabad School of Medical Sciences, Asadabad, Iran; 2 Endocrinology and Metabolism Research Center, Hormozgan University of Medical Sciences, Bandar Abbas, Iran; University of Chittagong, BANGLADESH

## Abstract

**Background:**

Bacterial vaginosis (BV) is the most common vaginal dysbiosis in fertile women, which is associated with side effects including the risk of premature birth. *Gardnerella vaginalis* (*G*. *vaginalis*) is a facultative anaerobic bacillus known as the main pathogen responsible for BV. In this study, using bioinformatics and immunoinformatics methods, a multi-epitope vaccine with optimal population coverage against BV caused by *G*. *vaginalis* was designed.

**Methods:**

Amino acid sequences of two important virulence factors (Vaginolysin and Sialidase) of *G*. *vaginalis* were retrieved from NCBI and UniProt databases. At first, three online servers ABCpred, BCPREDS and LBtope were used to predict linear B-cell epitopes (BCEs) and IEDB server was used for T cells. Then the antigenicity, toxicity, allergenicity were evaluated using bioinformatics tools. After modeling the three-dimensional (3D) structure of the vaccine by Robetta Server, molecular docking and molecular dynamics were performed. Finally, immune simulation and *in silico* cloning were considered effective for the design of vaccine production strategy.

**Results:**

In total, six epitopes of BCEs, eight epitopes from CD4^+^ and seven epitopes from CD8^+^ were selected. The designed multi-epitope vaccine was non-allergenic and non-toxic and showed high levels of antigenicity and immunogenicity. After the 3D structure was predicted, it was refined and validated, which resulted in an optimized model with a Z-score of -7.4. Molecular docking and molecular dynamics simulation of the designed vaccine revealed stable and strong binding interactions. Finally, the results of vaccine immunity simulation showed a significant increase in immunoglobulins, higher levels of IFN-γ and IL-2.

**Conclusion:**

According to the findings, the candidate multi-epitope vaccine has stable structural features. It also has the potential to stimulate long-term immunity in the host, but wet-lab validation is needed to justify it.

## Introduction

Bacterial vaginosis (BV) is the most common vaginal dysbiosis in women of reproductive age, which is the cause of several serious consequences for patients, including the risk of premature birth, pelvic inflammatory disease (PID), increased susceptibility to sexually transmitted infections (STI), and human immunodeficiency viruses (HIV) [1–4]. It has been estimated that the annual economic cost of treating bacterial vaginosis (with a prevalence of 10–30% worldwide) is approximately $5 billion [5]. This syndrome is characterized by clinical symptoms such as homogeneous, thin and white vaginal discharge, increased vaginal pH (pH > 4.5), fishy odor from vaginal secretions, and overgrowth of pathogenic anaerobic bacteria such as *Gardnerella vaginalis* (*G*. *vaginalis*) and *Prevotella bivia [6, 7]*. However, many women with BV are asymptomatic.

*G*. *vaginalis* is a facultative anaerobic Gram-variable bacterium that inhabits the normal flora of the women vagina [8]. This bacterium was described as the proposed cause of BV (isolated from cervical swabs and urine samples) by Gardner and Duke in 1955 [9]. Extensive research showed that *G*. *vaginalis* has key virulence factors such as vaginolysin (VLY) and sialidase, etc., which it uses to colonize and persist in the vaginal microenvironment [10]. VLY is a 57 kDa protein known to be the most important virulence factor of BV. VLY is a cholesterol-dependent cytolysin (CDC) that lyses sensitive cells such as vaginal epithelial cells by interacting with the CD59 molecule [11]. Studies have shown that vaginolysin is also packaged inside membrane vesicles of *G*. *vaginalis* [12]. The pH condition in vesicles has a significant effect on the production process of this virulence factor in a way that it is not produced in more acidic conditions [13].

Vaginal mucosa is a protective barrier consisting of sialoglycoproteins called mucins, which play a vital role in protective and defensive mechanisms against pathogens [14]. Sialidases have been identified as a major Gardnerella virulence factor that cleaves terminal sialic acid residues from human glycans [15]. In addition, sialidases play a critical role in the attachment, colonization, and dissemination of many other vaginal pathogens [16]. In *G*. *vaginalis* isolates, three homologs of sialidase, NanH1, NanH2 and NanH3 have been identified. Recently, studies have shown that sialidase activity is mainly higher in NanH2 and NanH3 than in NanH1 [17]. In general, targeting the sialidase activity of *G*. *vaginalis* can be an effective step in the prevention of BV [10].

Today, studies based on the design and manufacture of multi-epitope vaccines have made significant progress in the pharmaceutical industry. Guo et al. used an *in silico* and *in vivo* approach to predict HLA-I-restricted CTL epitopes on HPV16 E5, E6, and E7 proteins. Their findings showed that the predicted epitopes could induce antigen-specific IFN-γ secretion in mice and that prophylactic immunization with E5E6E7pep11 and CTB-Epi11E567 provided 100% protection against tumor growth in mice. Also, other studies have shown the efficacy of multi-epitope vaccines in vivo [18]. In addition, epitope-based vaccines against microbial pathogens have progressed to clinical trials in humans [19, 20] including Bionor Immuno’s HIV p24 gag peptide vaccine (Vacc-4X) and epitope-focused recombinant protein-based malaria vaccine (RTS,S/AS) which are in phase II and III clinical trials, respectively. Until now, these vaccines have been proven to be effective, safe and well tolerate in humans [19–23].

Bioinformatic tools have shown that they have the ability to design conserved sequences as suitable and practical vaccine candidates. Therefore, in this study, we designed a multi-epitope candidate vaccine against bacterial vaginosis in *G*. *vaginalis* using immunoinformatics design tools. It should be noted that so far no study has been performed on multi-epitope vaccines against BV in *G*. *vaginalis*.

## Materials and methods

In order to design a multi-epitope vaccine against bacterial vaginosis caused by *G*. *vaginalis*, the desired proteins were extracted and epitopes stimulating CD4^+^ and CD8^+^ T cells and B cells were selected. Then for validation, allergenicity, toxicity and physicochemical properties of all epitopes were done using different web servers. Three linkers AAY, GPGPG and KK were selected to bind the epitope of cytotoxic T cell (CTL), T-helper lymphocyte (HTL) and B cell lymphocyte (BCL), respectively. In addition, to evaluate stability and binding affinity, TLR4 receptor was docked by ligands using ClusPro 2.0 server. Finally, codon compatibility and *in silico* simulation studies were performed. In addition, the C-ImmSim server was used to describe the humoral and cellular profile of the mammalian immune system against the designed vaccine.

### Retrieval of protein sequences

In this study, two important proteins related to sialidase (accession number: PNL25438.1) and vaginolysin (entry: A0A2I1KNS6) were selected to design multi-epitope vaccines against BV caused by *G*. *vaginalis*. Protein sequences were retrieved from the National Center for Biotechnology Information (https://www.ncbi.nlm.nih.gov/protein/?term=) and the Universal Protein Resource (https://www.uniprot.org/) databases in FASTA format.

### Prediction of linear B cell epitopes

Linear B-cell epitopes (BCEs) are recognized as essential components of multi-epitope vaccines, which play a key role in the development of peptide vaccines and disease diagnosis [24–26]. We used three different servers, ABCpred, BCPREDS and LBtope, to obtain the best coverage of predicted BCEs [24, 27, 28]. ABCpred (http://crdd.osdd.net/raghava/abcpred/ABC_submission.html) server is designed based on a recurrent neural network to predict the BCEs in an antigenic sequence. This server is able to predict epitopes with 65.93% accuracy [24]. We used window lengths of 16 and 18 amino acids and threshold of 0.51 to predict BCEs. Another server used to predict linear BCEs was BCPREDS (http://ailab-projects2.ist.psu.edu/bcpred/) [27]. Due to the high accuracy of epitope prediction of this server, we considered window lengths of 16, 18, and 20 amino acids and specificity 0.75% to predict BCEs. The third server for predicting BCEs was LBtope (https://webs.iiitd.edu.in/raghava/lbtope/protein.php). In this server, various machine learning techniques such as Support Vector Machine, and K-Nearest Neighbor are used to distinguish between epitopes and non-epitopes [28].

### Identification of MHC-I binding epitopes

Prediction of MHC class I epitopes was performed using IEDB server (https://tools.iedb.org/mhci/) [29]. The parameter settings chosen for the MHC I server included: prediction method = NetMHCpan 4.1 EL, MHC source species = human, MHC allele = human leukocyte antigen (HLA) allele reference set (18 HLA-A, 32 HLA-B and 20 HLA-C), epitope length = 9mer. Finally, a score higher than 0.5 was considered to predict MHC-I epitopes.

### MHC class II binding prediction

The IEDB MHC-II (http://tools.iedb.org/mhcii/) binding prediction tool was used to predict T-helper cell epitopes. Parameters used to predict MHC II epitopes include: prediction method = NetMHCpan 4.1 EL (recommended epitope predictor-2023.09), MHC allele = HLA reference set (containing 27 alleles; HLA-DR, HLA-DP, HLA-DQ), epitope length = 15mer. Finally, adjusted rank < 2 was considered to predict MHC-II epitopes.

### Designed multi-epitope vaccine complex

All predicted HTL, CTL and B cell epitopes were joined by separate linkers. AAY (Ala-Ala-Tyr), GPGPG (Gly-Pro-Gly-Pro-Gly) and KK (Lys-Lys) linkers were used to fused CTL, HTL and BCL epitopes, respectively. The GPGPG linker plays a role in flexibility and induction of HTL responses [30]. The KK linker provides flexibility and spatial separation in protein structures [31]. On the other hand, the AAY linker, which acts as a proteasome cleavage site in mammalian cells, facilitates the formation of natural epitopes and increases the immunogenicity of the vaccine [30, 32]. To increase immunogenicity, cholera enterotoxin B subunit adjuvant (accession number: P01556) was attached to the vaccine’s N-terminal by the EAAAK linker.

### Physicochemical characteristics of multi-epitope vaccine

Physical and chemical properties are crucial for the stability and efficiency of proteins. Therefore, the ProtParam server (http://web.expasy.org/protparam/) was used to evaluate the physical and chemical properties of the final structure of the vaccine [33]. The instability index (II) of a protein indicates its stability of the protein. If this index is less than 40, it is predicted as stable, and otherwise (i.e. greater than 40), it indicates protein instability. The value of GRAVY was considered for the protein construct, where positive values of GRAVY indicate the hydrophobic nature of the protein and negative values mean its hydrophilicity [34–36]. Aliphatic index (AI) plays an important role in protein thermal stability [25]. Protein solubility is considered as an important feature in vaccine design, which is important in therapeutic application [37]. For this reason, in the present study, the Protein-Sol web server (https://protein-sol.manchester.ac.uk/) was used to predict protein solubility.

### Evaluation of antigenicity, allergenicity and toxicity of multi-epitope vaccine

We used VaxiJen (http://www.ddg-pharmfac.net/vaxijen/VaxiJen/VaxiJen_citation.html) server to predict protein antigenicity [38]. VaxiJen server was recognized as the first antigen prediction server. This server evaluates the ability to predict the antigenicity of a set of bacterial, viral and tumor protein data with 70–89% accuracy [38]. It should be noted that a threshold higher than 0.4 was chosen for the target organism. In addition, a score of more than 0.8 for ANTIGENpro server (https://scratch.proteomics.ics.uci.edu/) is considered as an antigenic index in this study [39]. On the other hand, the allergenicity of predicted epitopes was performed using AllerTOP v.2.0 server (http://www.ddg-pharmfac.net/AllerTOP/). The reason for choosing the AllerTOP v.2.0 server is its accuracy (88.7%) and high sensitivity (94%) compared to other allergen prediction servers. The ToxinPred server (http://crdd.osdd.net/raghava/toxinpred/multi_submit.php) was used to predict the toxicity of peptides [40].

### Population coverage of epitopes

The frequency of distribution of distinct HLA alleles varies in different geographic regions and ethnicities. Therefore, the IEDB population coverage analysis tool (http://tools.iedb.org/population/) was used to assess the population coverage of vaccine candidates [41]. In the present study, selected epitopes of CTL and HTL as well as their MHC alleles were investigated in several continents.

### Secondary and tertiary structure prediction of the vaccine construct

Protein secondary structure is one of the most important issues in bioinformatics and is also an important step towards clarifying the 3-dimensional (3D) structure and its function [42]. In this regard, the PSIPRED 4.0 server (http://bioinf.cs.ucl.ac.uk/psipred/) was used to predict the secondary structure of the designed multi-epitope vaccine with an accuracy of 84.2% [43]. Robetta server (https://robetta.bakerlab.org/) was used to predict the 3D structure of the protein [44]. This server uses automated tools (CAMEO; Continuous Automated Model Evaluation) to analyze and predict protein structure with a de novo modeling approach.

### Evaluation of refinement and validation features

The 3D protein model was refined using the GalaxyRefine web server [45]. MolProbity provides structure validation and predicted model quality in 3D structures of proteins and nucleic acids [46]. Typical scores for experimental structures in this index range from 1 to 2. RMSD is one of the most useful quantitative measures used with an empirical crystallographic source for structural comparisons between different conformations of a molecule [45, 47]. Due to protein flexibility, the RMSD score varies between 0 and 1.2 Angstroms (Å). In addition, a lower value of RMSD indicates better stability [48, 49].

UCLA-DOE LAB and ProSA-web were used to evaluate the different stereochemical parameters of the protein structure and predict the structural quality of the selected model [50, 51]. The UCLA-DOE LAB server has various subsets such as PROCHECK, ERRAT and Verify-3D for 3D structure validation. Ramachandran plot was analyzed by PROCHECK section. This plot shows the statistical distribution of the composition of backbone dihedral angles φ and ψ as well as the percentage and number of residues. Different atoms in the structure of proteins are randomly distributed relative to each other, which requires the investigation of statistical methods to correct them [50]. Therefore, ERRAT tool was used to obtain the best distribution of atoms. On the other hand, Verify-3D analyzes the accuracy of a 3D protein model with its amino acid sequence [52, 53].

The ProSA (Protein Structure Analysis) server is used in the modification and validation of protein structures. One of the features of this server is checking the overall quality of the model and the energy deviation of the entire structure from different sources (X-ray, NMR) by showing the z-score plot [51].

### Molecular docking

Molecular docking predicts the binding affinity of ligands to receptor proteins and is recognized as an important tool for drug discovery [54]. Molecular docking between designed multi-epitope vaccine and Toll-Like receptor 4 (TLR4) (PDB ID: 2Z62) was performed with ClusPro server (https://cluspro.org). This server performs various computational steps such as rigid body connection, clustering of 1000 low energy structures, and refinement energy minimization. The LigPlot + software was used to analyze the bindings formed between the vaccine complex and TLR4.

### Molecular dynamic simulation

In the selected model, intense steric clashes may occur between residues due to the unusual overlap of nonbonding atoms. Therefore, molecular dynamics (MD) simulations were conducted using GROMACS 2021.5 [55]. The protein topology parameters were generated with the Amber99.sb force field. In addition, the protein was placed in a dodecahedron box, and the TIP3P water model was selected. To neutralize the system, an appropriate number of Cl or Na ions were added to replace some of the solvent molecules and energy minimization was carried out in a total of 100000 steps. The MD simulation began with NVT (constant volume and temperature) and NPT (constant pressure and temperature) conditions for one nanosecond. Finally, the MD run lasted for 100 nanoseconds (ns).

### Normal mode analysis (NMA)

Normalized mode analysis (NMA) is a technique that can be used to describe the flexible states accessible to a protein about its equilibrium position [49]. For this reason, iMODS server (http://imods.Chaconlab.org) was used to predict such situations [56]. This server uses NMA in internal coordinates (dihedral) which can predict collective functional motions of biological macromolecules. This server presents the results in the form of graphs by calculation of deformability, B-factor, eigenvalues, variance, covariance map and elastic network.

### Immune simulation

Multi-epitope vaccine immunological simulations were performed by the C-IMMSIM server [57]. This server can simulate the immune response generated by the mammalian thymus (T cells), bone marrow (lymph and bone marrow cells), and a lymphatic organ. In other words, the C-IMMSIM server represents the main classes of lymphoid cells Th, CTL, B lymphocytes, antibody-producing plasma cells (PLB) and myeloid lineage [macrophages (M) and dendritic cells]. To create an efficient and long-term immune response, three doses of the vaccine with time intervals of 1, 84 and 168 days were considered. Also, the simulation volume and simulation steps were set to 10 and 1100 respectively.

### Codon optimization and in silico cloning

Java Codon Compatibility Tool (JCat) server was used for reverse translation and codon optimization of the designed vaccine construct in *Escherichia coli* k-12 strain [58]. Two important features in the output of this server include guanine-cytosine (GC: 30% to 70%) content and codon compatibility index (CAI> 0.8) score, which evaluate protein expression levels. In addition, the candidate vaccine was cloned into pET-28a (+) plasmid using SnapGene software (version 5.2.3). The EcoRI (GAATTC) and BamHI (GGATCC) restriction sites were added to the N-terminal and C-terminal of the optimized gene respectively.

### Analysis of the vaccine MRNA

Today, significant improvements have been achieved from the success of mRNA vaccines [59]. We used RNAfold and Mfold v2.3 online servers to predict the secondary structure of vaccine mRNA [60].

## Results

### Retrieval of protein sequences

Two protein sequences with different amino acid length, sialidase (907 aa) and vaginolysin (541 aa) were retrieved in FASTA format.

### BCEs prediction

Based on ABCpred, BCPREDS and LBtope servers, a total of six epitopes were selected with criteria such as antigenic, non-allergenic and non-toxic (Table 1). Common epitopes in two or three high-scoring servers were selected.

**Table 1:** Prediction of B cell epitopes based on ABCpred, LBtop and BCPREDS servers.

Protein	Peptide	BCPREDS	LBtop	ABCpred	Antigenicity	Allergenicity	Toxicity
**Vaginolysin**	NRGVDNKRPPVYVSNVAYGR	0.993	76.44	-	0.6545	NON-ALLERGEN	Non-Toxin
	ETIENKFSSDSFNKNGEF	-	61.29	0.81	0.6812	NON-ALLERGEN	Non-Toxin
**Sialidase A**	NSENADNADCIAFANA	0.989	-	0.68	0.4860	NON-ALLERGEN	Non-Toxin
	PNPEEIANSSTKAQPD	0.979	61.18	0.70	0.7580	NON-ALLERGEN	Non-Toxin
	NSHSNQTCDSSTWNIWKS	-	68.80	0.91	0.6968	NON-ALLERGEN	Non-Toxin
	NESGINRKDKNEEINQNEGIIG	0.95	64.74	-	0.8073	NON-ALLERGEN	Non-Toxin

### Identification of MHC-I and MHC-II binding epitopes

MHC-I (9 mer) and MHC-II (15 mer) binding epitopes predicted by IEDB recommended methods for available alleles (MHC-I; HLA-A, HLA-B, and HLA-C) and (MHC-II; HLA-DR, HLA-DQ, and HLA-DP) was performed by the IEDB server. Eight epitopes of MHC-I and seven epitopes of MHC-II were selected for the final vaccine construct (Tables 2 and 3). It should be noted that all selected epitopes were evaluated in terms of antigenic, non-allergenic and non-toxic.

**Table 2:** Most probable predicted epitopes with MHC class I alleles from IEDB analysis tool.

Protein	Peptide sequence	Allele	Score	Antigenicity	Allergenicity	Toxicity
**Vaginolysin**	RVYPGALFR	HLA-A*31:01	0.987597	0.4368	NON-ALLERGEN	Non-Toxin
		HLA-A*03:01	0.985599			
		HLA-A*11:01	0.978472			
		HLA-A*68:01	0.907007			
		HLA-A*30:01	0.778029			
		HLA-A*33:01	0.527506			
	YIETKVSSY	HLA-A*01:01	0.860917	0.6501	NON-ALLERGEN	Non-Toxin
		HLA-B*15:01	0.669158			
	KQTQIVNFK	HLA-A*11:01	0.648391	0.4803	NON-ALLERGEN	Non-Toxin
		HLA-A*03:01	0.616057			
**Sialidase A**	KEAEASTSL	HLA-B*40:01	0.995514	1.2509	NON-ALLERGEN	Non-Toxin
		HLA-B*44:03	0.711046			
		HLA-B*44:02	0.640662			
	SEKECATKW	HLA-B*44:03	0.992693	0.9614	NON-ALLERGEN	Non-Toxin
		HLA-B*44:02	0.991498			
	REHDFAITY	HLA-B*44:03	0.98652	1.1129	NON-ALLERGEN	Non-Toxin
		HLA-B*44:02	0.968665			
		HLA-B*40:01	0.709703			
		HLA-B*15:01	0.654605			
	RSTDGGKTW	HLA-B*58:01	0.987509	1.6403	NON-ALLERGEN	Non-Toxin
		HLA-B*57:01	0.979395			
	TPSSEIKKY	HLA-B*35:01	0.922394	0.9043	NON-ALLERGEN	Non-Toxin
		HLA-B*53:01	0.694672			

**Table 3:** Predicted epitopes of MHC class II alleles.

Protein	Peptide sequence	Allele	Antigenicity	Allergenicity	Toxicity
**Vaginolysin**	KQTYYTVSVDAPDSP	HLA-DQA1*03:01/DQB1*03:02	0.4139	NON-ALLERGEN	Non-Toxin
		HLA-DRB1*04:05			
		HLA-DQA1*05:01/DQB1*02:01			
		HLA-DRB1*04:01			
		HLA-DRB1*09:01			
	SKSTDFQAAVEAAIK	HLA-DRB1*09:01	0.8539	NON-ALLERGEN	Non-Toxin
		HLA-DRB1*07:01			
	IENKFSSDSFNKNGE	HLA-DPA1*03:01/DPB1*04:02	1.0451	NON-ALLERGEN	Non-Toxin
		HLA-DPA1*01:03/DPB1*04:01			
**Sialidase A**	LFDTGYAGSRSYRIP	HLA-DRB1*09:01	0.5590	NON-ALLERGEN	Non-Toxin
		HLA-DRB1*07:01			
	SGTLHIQFRLRGPGQ	HLA-DRB1*11:01	1.2897	NON-ALLERGEN	Non-Toxin
		HLA-DRB1*08:02			
	SEIKKYSENESGINR	HLA-DRB1*15:01	0.7169	NON-ALLERGEN	Non-Toxin
		HLA-DRB3*02:02			
	TGYAGSRSYRIPSLV	HLA-DRB1*09:01	0.7502	NON-ALLERGEN	Non-Toxin

### Physicochemical characteristics of multi-epitope vaccine

**Fig 1:**
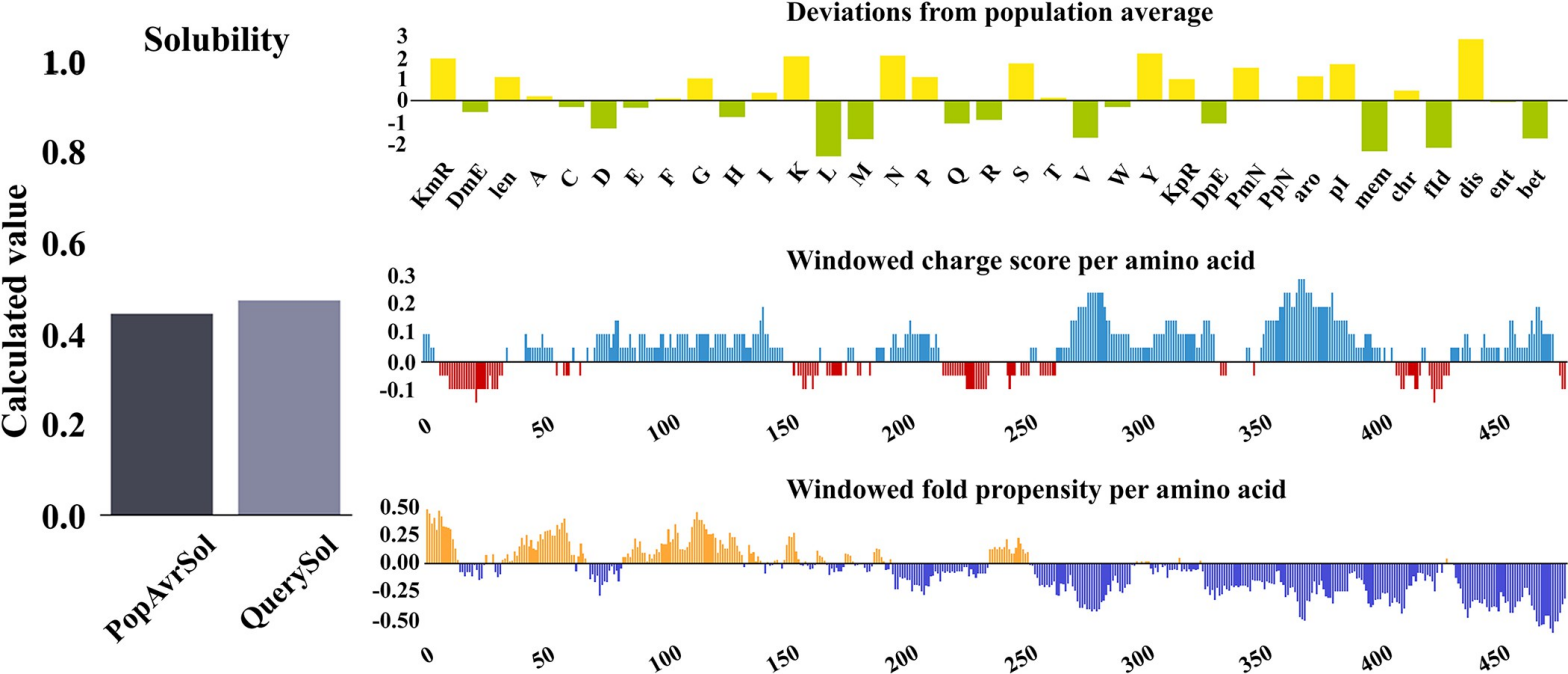
According to the QuerySol server, the solubility of the final construct was 0.481.

Based on the results of the ProtParam tool, the final construct of the designed multi-epitope vaccine containing 484 amino acids and its molecular weight of 52.7 kDa was determined. The theoretical PI of the vaccine was calculated to be 9.28. There were 49 negatively charged residues and 65 positively charged residues. The vaccine construct was composed of 7338 atoms, and its chemical formula was C2438H3779N685O753S9. The estimated half-life of the vaccine in mammalian reticulocytes (in vitro) is 30 hours, in yeast (in vivo) approximately >20 hours, and in *Escherichia coli* (in vivo) >10 hours, which shows the stability of the vaccine and its effectiveness in different biological environments. The AI index was 57.58 and the GRAVY index was -0.726, which indicates the thermally stable nature and effective interaction of the vaccine with water. In addition, the instability index of 35 was calculated, which showed that the candidate vaccine has good stability. On the other hand, the solubility of the vaccine structure according to QuerySol server was 0.481 (Fig 1).

### Multi-epitope vaccine construction

A combination of different epitopes, including six BCEs, eight MHC-I epitopes, and seven MHC-II epitopes by AAY, GPGPG and KK linkers, were considered for the multi-epitope vaccine. To enhance the immunogenicity of the construct, cholera enterotoxin B subunit adjuvant (MIKLKFGVFFTVLLSSAYAHGTPQNITDLCAEYHNTQIYTLNDKIFSYTESLAGKREMAIITFKNGAIFQVEVPGSQHIDSQKKAIERMKDTLRIAYLTEAKVEKLCVWNNKTPHAIAAISMAN) was added by EAAAK linker.

### Evaluation of antigenicity, allergenicity and toxicity of multi-epitope vaccine

The antigenic result of the vaccine designed by VaxiJen and ANTIGENpro servers was predicted as 0.7642% and 0.9364%, respectively, which indicates its high antigenicity. Also, the candidate vaccine was examined in terms of allergenicity and toxicity. Based on the results of the vaccine model, it is non-allergenic and non-toxic in both AllerTOP and ToxinPred servers, respectively.

### Secondary and 3D structure prediction of the vaccine construct

**Fig 2:**
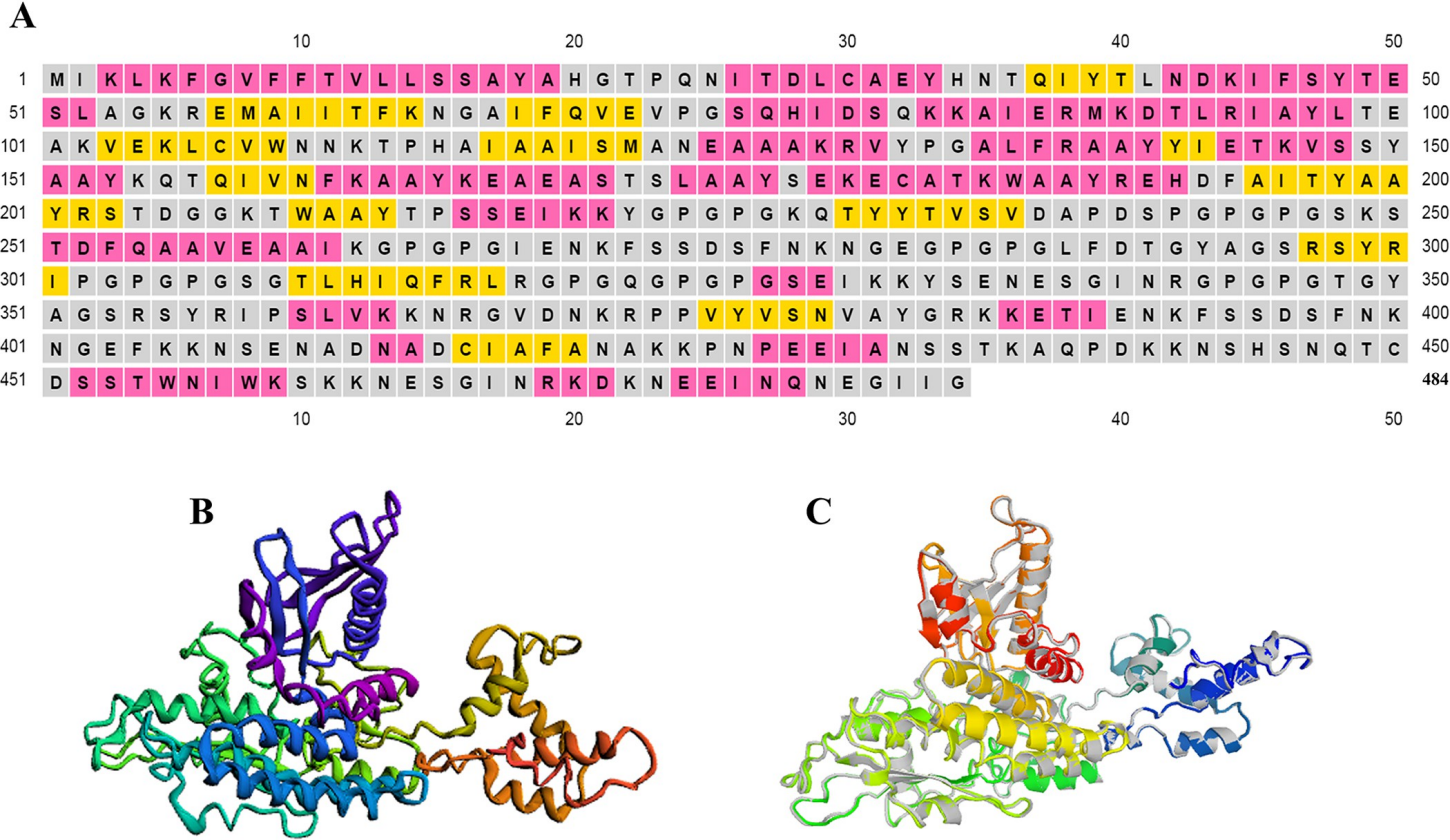
Displays the second and 3D of the designed multi-epitope vaccine. A) Secondary structure of the final construct by PSIPRED 4.0 server showed that the vaccine candidate contains 33.26% α-helix, 14.46% β-strand, 52.28% random coil. B) 3D structure of multi-epitope vaccine selected by Robetta server as the best model. C) 3D structure of the multi-epitope vaccine after refinement.

Secondary structure results showed that the candidate vaccine contained 33.26% α-helix, 14.46% β-strand, 52.28% random coil (Fig 2A). Among the five models predicted by the Robetta server, model 3 (Fig 2B) was selected.

### Population coverage

**Fig 3:**
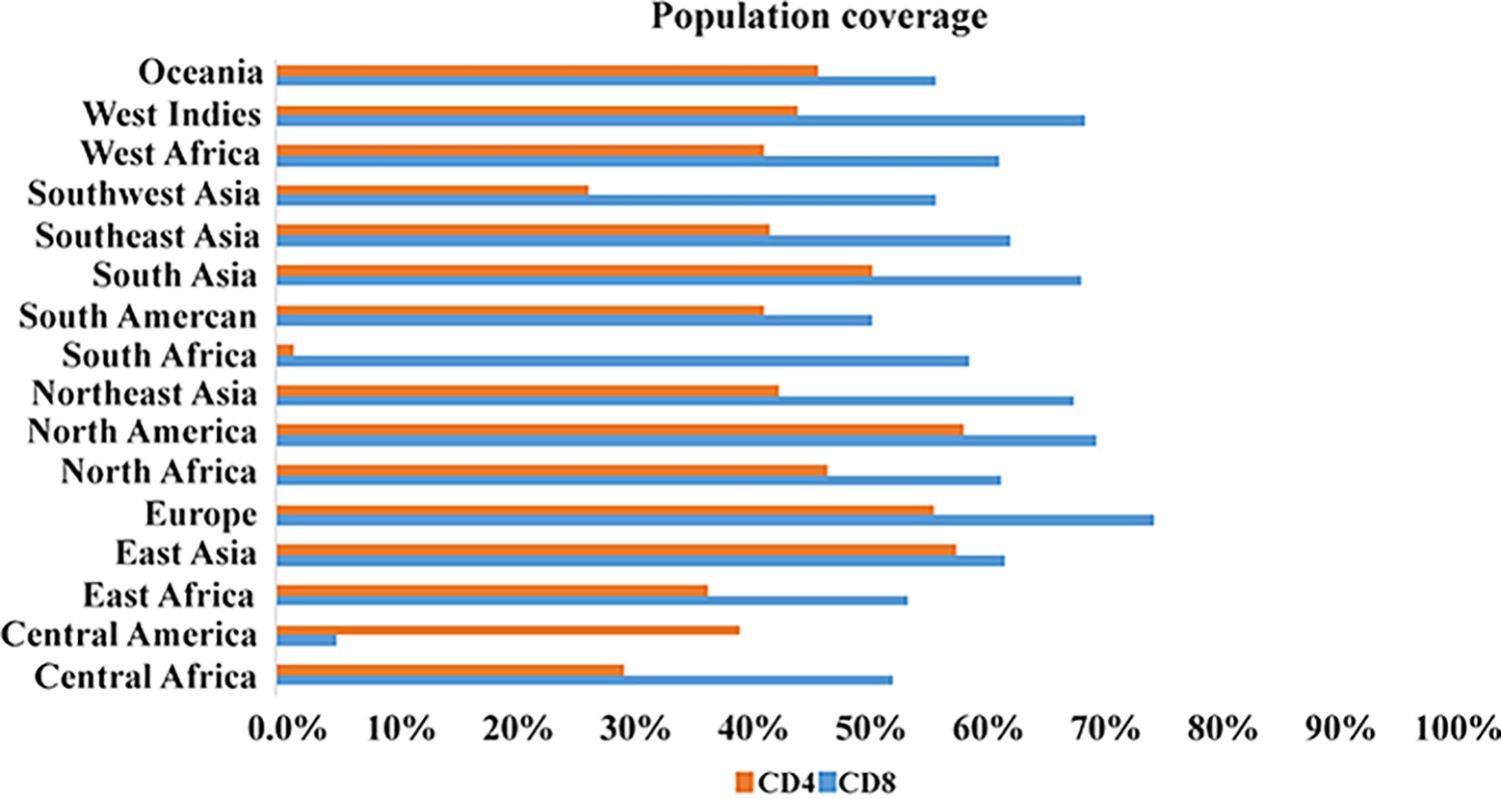
Results from worldwide population coverage rates (CD8^+^ and CD4^+^ T cell epitopes).

In the present study, the coverage of CD8^+^ and CD4^+^ T cell populations in different geographical regions of the world was investigated (Fig 3). The results showed that among eight CD8^+^ T cell epitopes, the highest coverage was in Europe (82.39%), North America (77.03%), West India (75.96%), and South Asia (75.47%). After that, Northeast Asia (74.78%), Southeast Asia (68.88%), East Asia (68.37%), North Africa (68.1%), West Africa (67.88%), South Africa (65.01%), Oceania (61.96%), Southwest Asia (61.93%), East Africa (59.28%), Central Africa (57.84%), South America (56.01%) provided other coverage. The lowest coverage was for the Central America region (5.69%).

The highest coverage for CD4^+^ T-cell epitopes was found in North America (64.51%), East Asia (63.82%), and Europe (61.76%). Other results were reported in North Africa (59.37%), South Asia (55.91%), Oceania (50.81%), West Indies (48.42%), Northeast Asia (47.25%), Southeast Asia (46.39%), South America (45.78%), West Africa (45.77%), Central America (43.57%), East Africa (40.56%), Central Africa (32.69%), and Southwest Asia (29.33%). South Africa (1.8%) had the lowest population coverage.

### Evaluation of refinement and validation features

After predicting the final 3D structure of the vaccine designed by the Robetta server, we used the GalaxyRefine server to increase the quality of the final vaccine structure. This server provides 5 models for the desired protein structure quality. Model number 3 (Fig 2C) including GDT-HA score 0.9851, RMSD score 0.300, MolProbity score 2.121, Clash score 13.2 and Ramachandran score 91.8 was selected (Table 4). This finding shows that the refined model is of good quality.

**Table 4:** Quality scores of 5 models predicted by GalaxyRefine server.

Model	GDT-HA	RMSD	MolProbity	Clash score	Poor rotamers	Rama favored
Initial	1.0000	0.000	1.771	4.4	0.0	89.8
MODEL 1	0.9841	0.310	2.127	11.0	1.2	91.6
MODEL 2	0.9856	0.292	2.099	12.6	0.5	92.0
MODEL 3	0.9851	0.300	2.121	13.2	0.5	91.8
MODEL 4	0.9846	0.303	2.042	10.2	0.2	91.2
MODEL 5	0.9781	0.318	2.073	11.2	0.2	91.4

**Fig 4:**
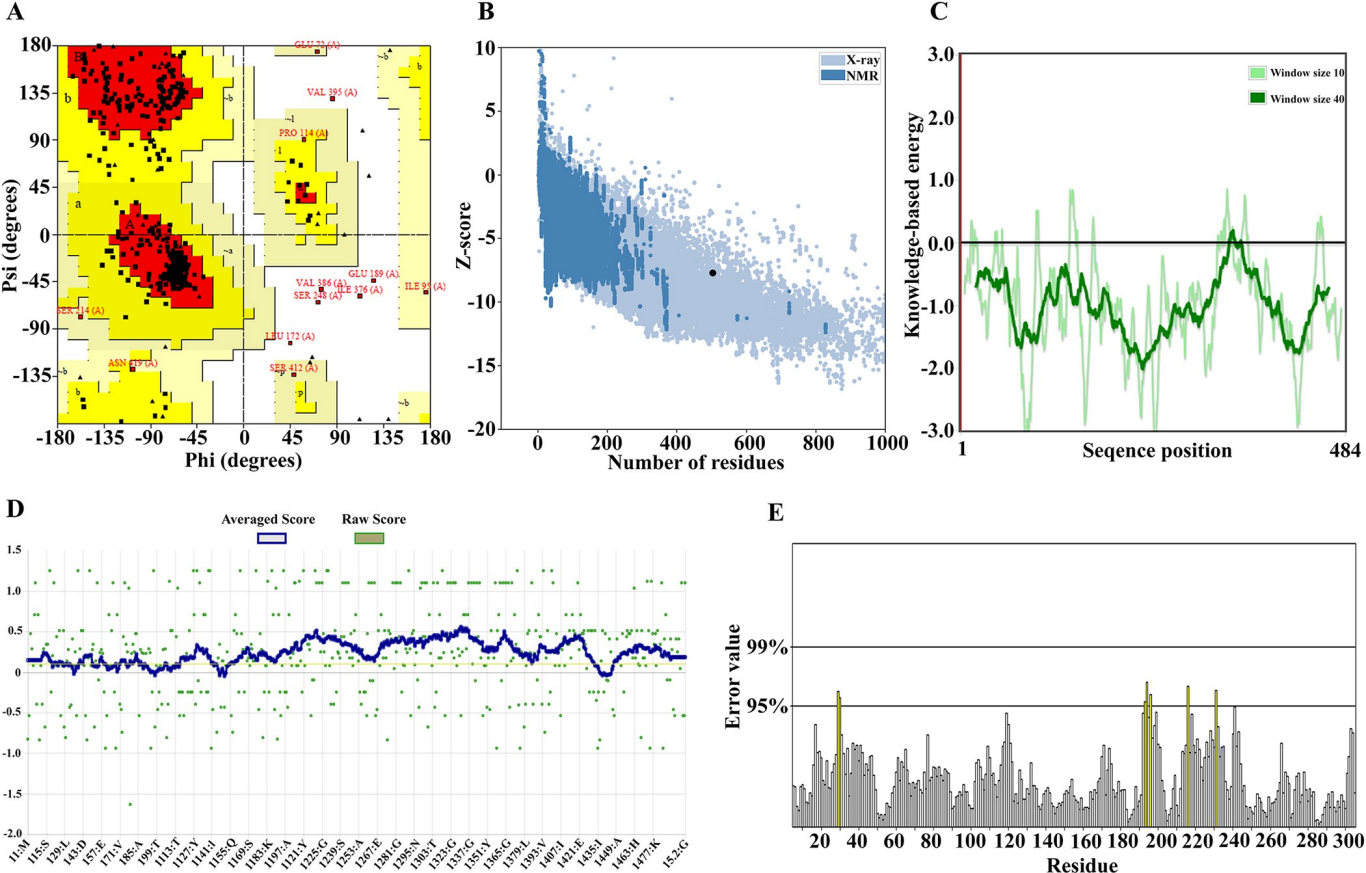
Evaluation of refinement and validation features. A) The statistics of the Ramachandran chart show the most favorable region, additionally allowed, generously and disallowed (outlier) area with 87.0%, 10.4%, 1.2% and 1.4%, respectively. B) The Z-score of the refined model is − 7.4. C) The quality of the local model. D) According to VERIFY 3D, the predicted model’s 84.26%of the residues have averaged 3D-1D score ≥ 0.2. E) Validation of the vaccine structure was reported by the ERRAT server with a score of 95.335.

Based on the UCLA-DOE LAB server, three parameters of PROCHECK, ERRAT, and Verify-3D were considered to evaluate the validity of the vaccine. The findings from the Ramachandran diagram created by the PROCHECK server showed that 87.0%, 10.4%, 1.2% and 1.4% of protein residues were located in the most favored region, additional allowed, generously and disallowed (outlier) area of the final vaccine, respectively (Fig 4A). The score of ERRAT, Verify-3D and Z-score was 95.335, 84.26% and -7.53, respectively, which indicates the good quality of the designed 3D model (Fig 4B–4D). Fig 4E showed the quality of the local drawing model. The negative values in this graph indicated that there is no error in the structure of the model.

### Molecular docking between designed vaccine with TLR4

**Fig 5:**
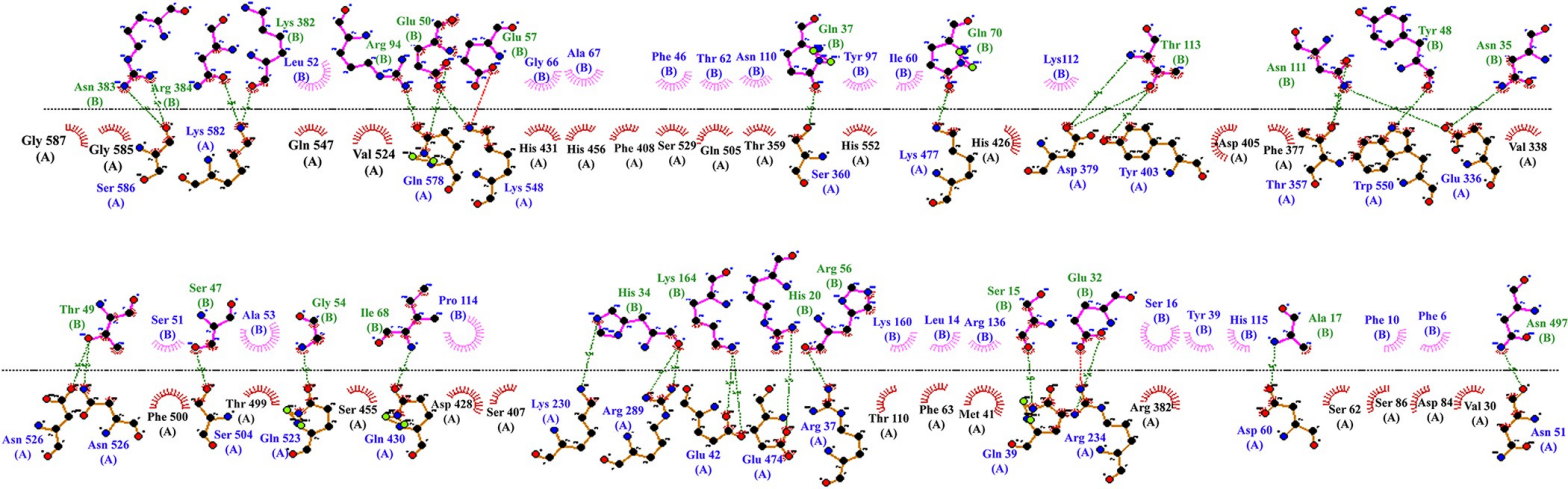
Representation of interacting residues between vaccine docked with TLR4. The Ser586, Lys582, Gln578, Lys548, Ser360, Lys477, Asp379, Tyr403, Thr357, Trp550, Glu336, Asn526, Ser504, Gln523, Gln430, Lys230, Arg289, Glu42, Glu474, Arg37, Gln39, Arg234, Asp60, Asn51 residues from chain A of the vaccine were bound to Asn383, Arg384, Lys382, Arg94, Glu57, Gln37, Gln70, Thr113, Asn111, Tyr48, Asn35, Thr49, Ser47, Gly54, Ile68, His34, Lys164, His20, Arg56, Ser15, Glu32, Ala17, Asn497 residues from chain B by hydrogen bonds.

Molecular docking, which is known as one of the essential aspects of in silico drug development, has a tendency to bind ligands to receptor proteins [61]. The energy score obtained for the best vaccine-TLR4 binding from the ClusPro v2.0 server was -937.1 (Table 5), indicating a strong binding affinity to TLR4. In this regard, the interaction between vaccine structure and TLR4 was established by LigPlot + software (Fig 5). Hydrogen bonds were obtained with DIMPLOT program. DIMPLOT was shown, Ser586, Lys582, Gln578, Lys548, Ser360, Lys477, Asp379, Tyr403, Thr357, Trp550, Glu336, Asn526, Ser504, Gln523, Gln430, Lys230, Arg289, Glu42, Glu474, Arg37, Gln39, Arg234, Asp60, Asn51 residues from chain A of the vaccine were bound to Asn383, Arg384, Lys382, Arg94, Glu57, Gln37, Gln70, Thr113, Asn111, Tyr48, Asn35, Thr49, Ser47, Gly54, Ile68, His34, Lys164, His20, Arg56, Ser15, Glu32, Ala17, Asn497 residues from chain B by hydrogen bonds.

**Table 5:** Top models of docked complexes of designed vaccine with TLR4.

Cluster	Members	Representative	Weighted Score
**0**	60	Center	-734.6
		Lowest Energy	-845.9
**1**	52	Center	-740.3
		Lowest Energy	-872.5
**2**	39	Center	-685.2
		Lowest Energy	-773.6
**3**	32	Center	-669.1
		Lowest Energy	-770.9
**4**	30	Center	-666.0
		Lowest Energy	-813.8
**5**	29	Center	-670.4
		Lowest Energy	-937.1
**6**	29	Center	-722.5
		Lowest Energy	-775.3
**7**	28	Center	-639.1
		Lowest Energy	-724.3
**8**	27	Center	-725.0
		Lowest Energy	-822.2
**9**	27	Center	-854.9
		Lowest Energy	-854.9
**10**	26	Center	-725.8
		Lowest Energy	-725.8

### MD simulation

**Fig 6:**
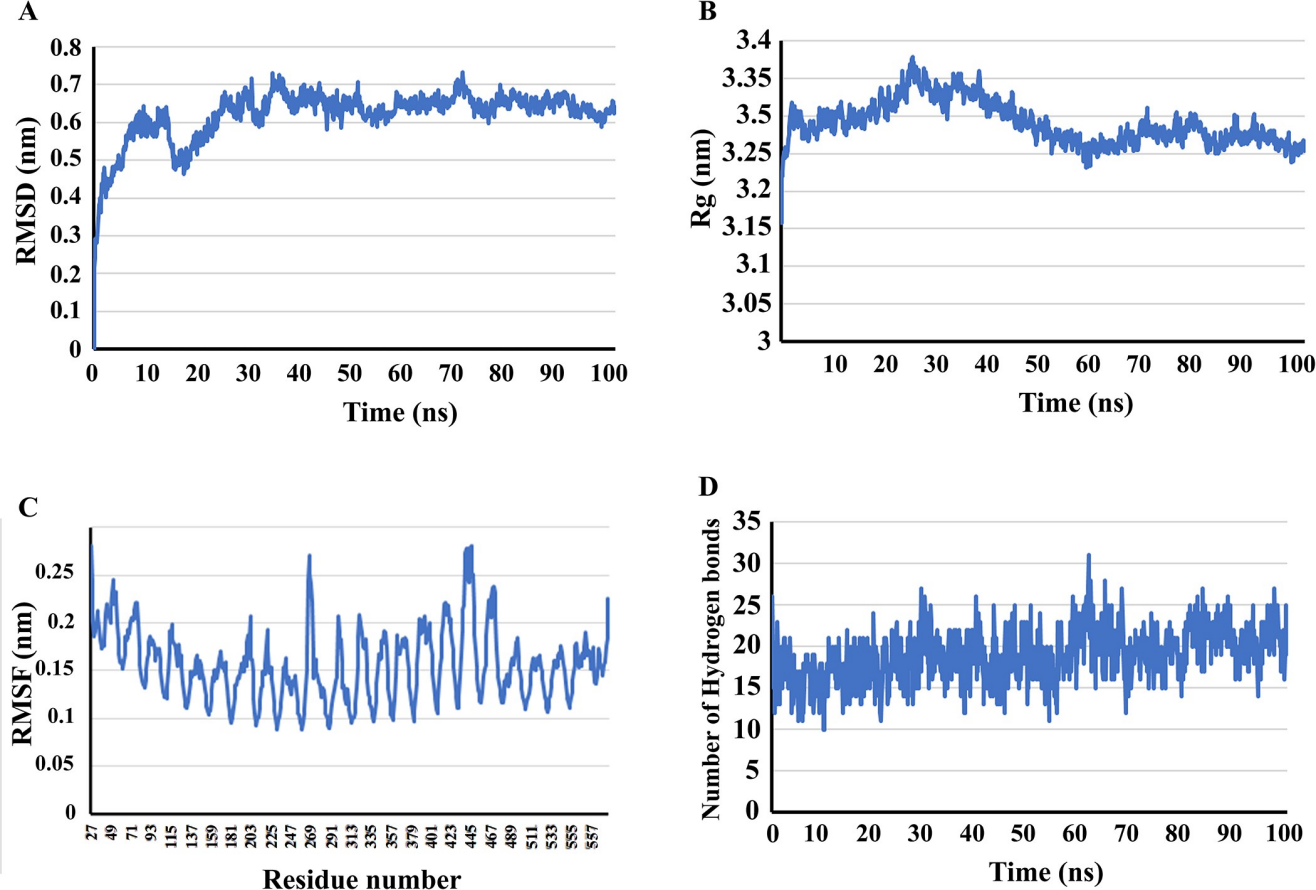
The final construct of the molecular dynamics simulation vaccine with GROMACS software. Comparison of changes in RMSD (A), Rg (B), RMSF (C), and (D) H-bond values of protein in interaction with TLR4.

Dynamics simulations at the molecular level were conducted using Gromacs 2021.5 software to investigate the stability of the vaccine-TLR4 complex during 100 ns. The backbone RMSD of the vaccine-TLR4 complex indicated that structural deviations within the complex stabilized after 30 ns. The backbone RMSD of the vaccine-TLR4 complex showed that the structural deviations in the complex stabilized after 30 ns. As illustrated in Fig 6A, the RMSD deviation was almost 0.1 nm for the last 70 ns. The RMSD data analysis concluded that the bounded residues between vaccine construct and TLR4 resulted in the stabilization of the interacted structure.

The compactness of the dynamically simulated structure was evaluated using a radius of gyration plot (Fig 6B). In the first 30 ns, the radius of gyration varied between 3.27 and 3.37 nm. After that, a decrease of almost 0.14 nm was observed until 60 ns. In the final 40 ns of the simulation phase, the reduced deviation and the relatively simple curve indicate improved compactness and stability. On the other hand, as shown in Fig 6C, RMSF was regarded the backbone of the vaccine-TLR4 complex. The RMSF analysis revealed fluctuations of nearly 0.2 nm in the residues of the structure, with the exception of residues 267 and 438 to 441. This indicates that the amino acids remained stable throughout the simulation.

The number of hydrogen bonds between vaccine construct and TLR4 were analyzed during simulations. As illustrated in Fig 6D, a maximum of 31 and a minimum of 10 hydrogen bonds were observed during the 100 ns molecular dynamics simulation, resulting in an average of 19 hydrogen bonds between vaccine construct and TLR4.

**Fig 7:**
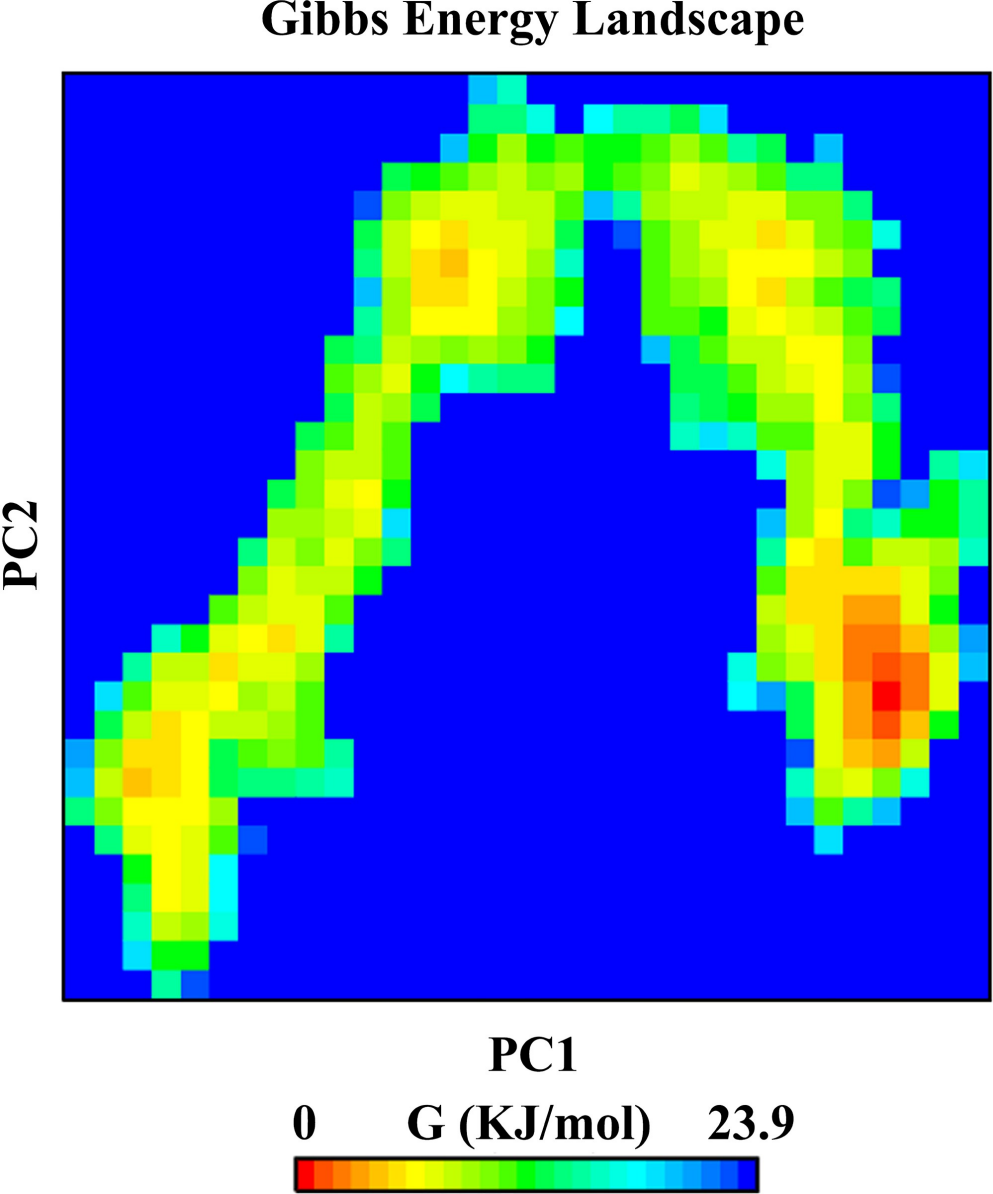
The Gibbs free energy landscape for the vaccine–TLR4 complex. Red lines represent lower energy levels, while dark blue represents higher energy levels.

The Gibbs free energy landscape (FEL) was calculated using the projections of the first (PC1) and second (PC2) eigenvectors. The two-dimensional FEL plot for the vaccine construct backbone in complex with the TLR4 is displayed in Fig 7. In the accompanying free energy contour map, red signifies lower energy levels, while dark blue represents higher energy levels.

### NMA evaluation of the vaccine-receptor complex

**Fig 8:**
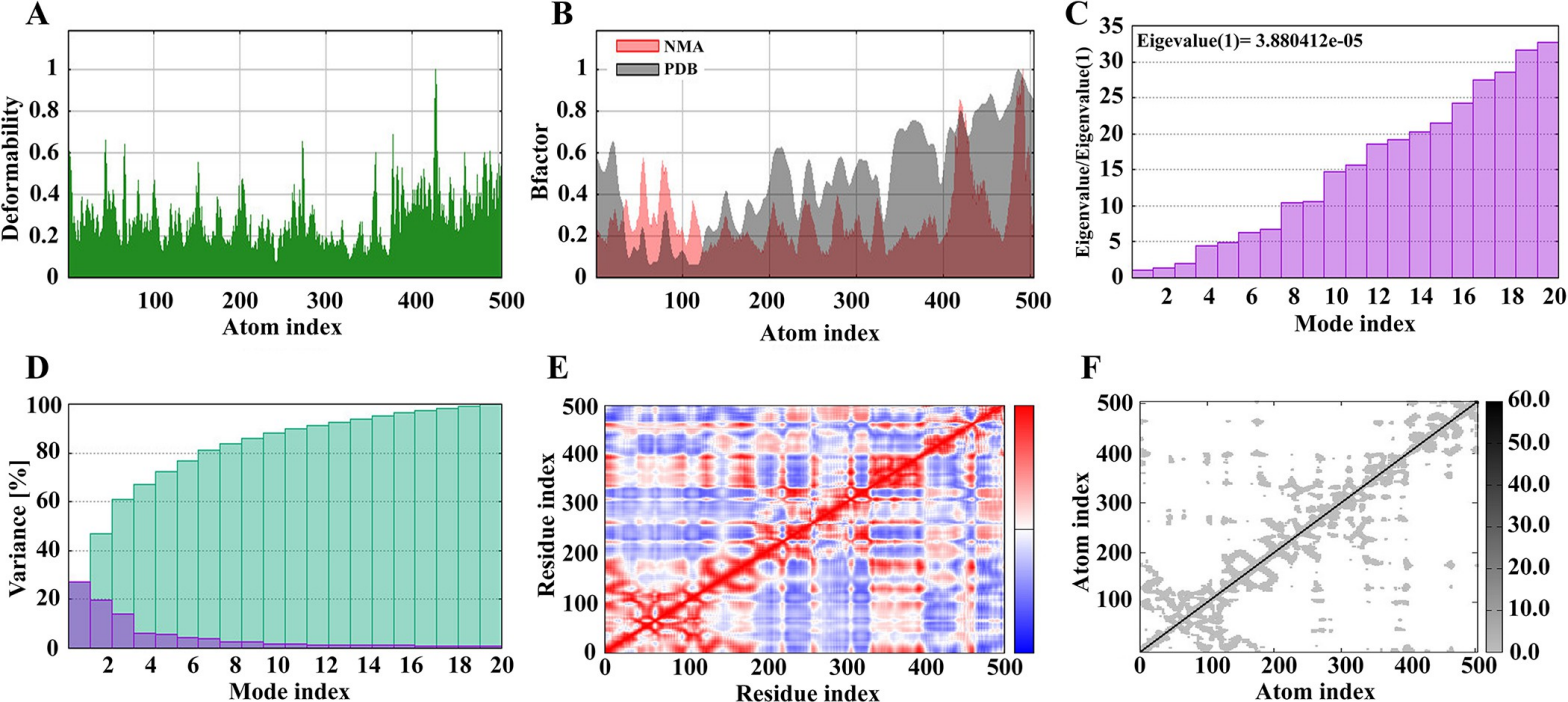
The molecular dynamics simulation of the vaccine–TLR4 complex.

The findings obtained from iMODS server are shown in Fig 8. Fig 8A shows regions of high deformability and a stable binding. Fig 8B shows the small fluctuations in atomic displacements to establish equilibrium. Fig 8C shows the eigenvalue determined for the set, which was 3.880412 e-07. This value indicate the energy required to deform the structure. Fig 8D shows the variance plot of the complexes. This graph has an inverse relationship with eigenvalues and highlights individual and cumulative variances in red and green, respectively. Fig 8E shows a covariance matrix map of the interaction between pairs of residues. Fig 8F shows the elastic network model for the stiffness of the protein complex. In this graph, the gray points represent stiffer areas, whose intensity is directly related to stiffness.

### Immunity simulation of multi-epitope vaccine

**Fig 9:**
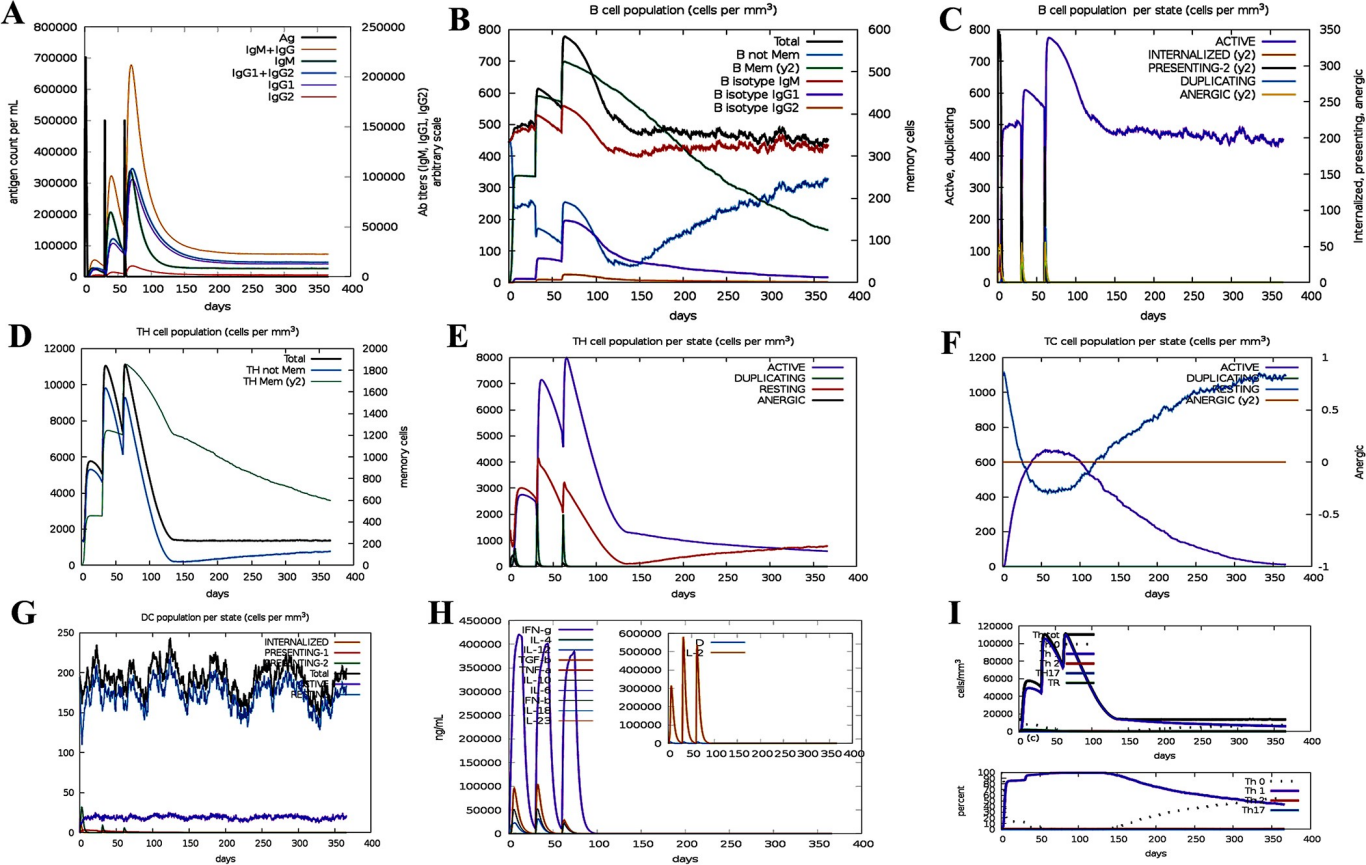
Immunity simulation of multi-epitope vaccine. A) The results showed that the level of IgM increased in the initial response, but in the second and third responses, the levels of IgM + IgG, IgG1 + IgG2, IgG1 and IgG2 were significantly higher than the first response. B) B cell population after three vaccine injections. C) Population per state of B-cell. D-F) The level of CTL/HTL cell population and memory cells (TCs) also increased significantly during vaccination. G) Dendritic cells increased with each exposure. H) High levels of IFN-γ and IL-2 were significantly increased after exposure. I) Finally, according to Fig 7I, Th1 was significantly increased.

Immunostimulation of candidate vaccine was performed by C-IMMSIM v10.1 web server. The results of this server showed that the IgM level increased in the initial response, but in the second and third responses, the levels of IgM + IgG, IgG1 + IgG2, IgG1 and IgG2 were significantly higher than the first response (Fig 9A). Also, B cell isotypes with high-stability were identified (Fig 9B). Fig 9C shows increased cell proliferation in B cells as well as antigen presentation after vaccination. On the other hand, the level of CTL/HTL cell population and memory cells (TCs) also increased significantly during vaccination (Fig 9D–9F). Dendritic cells (DCs) increased with each exposure (Fig 9G). High levels of IFN-γ and IL-2 were significantly increased after exposure (Fig 9H). Finally, according to Fig 9I, Th1 was significantly increased.

### Codon adaptation and in silico cloning

**Fig 10:**
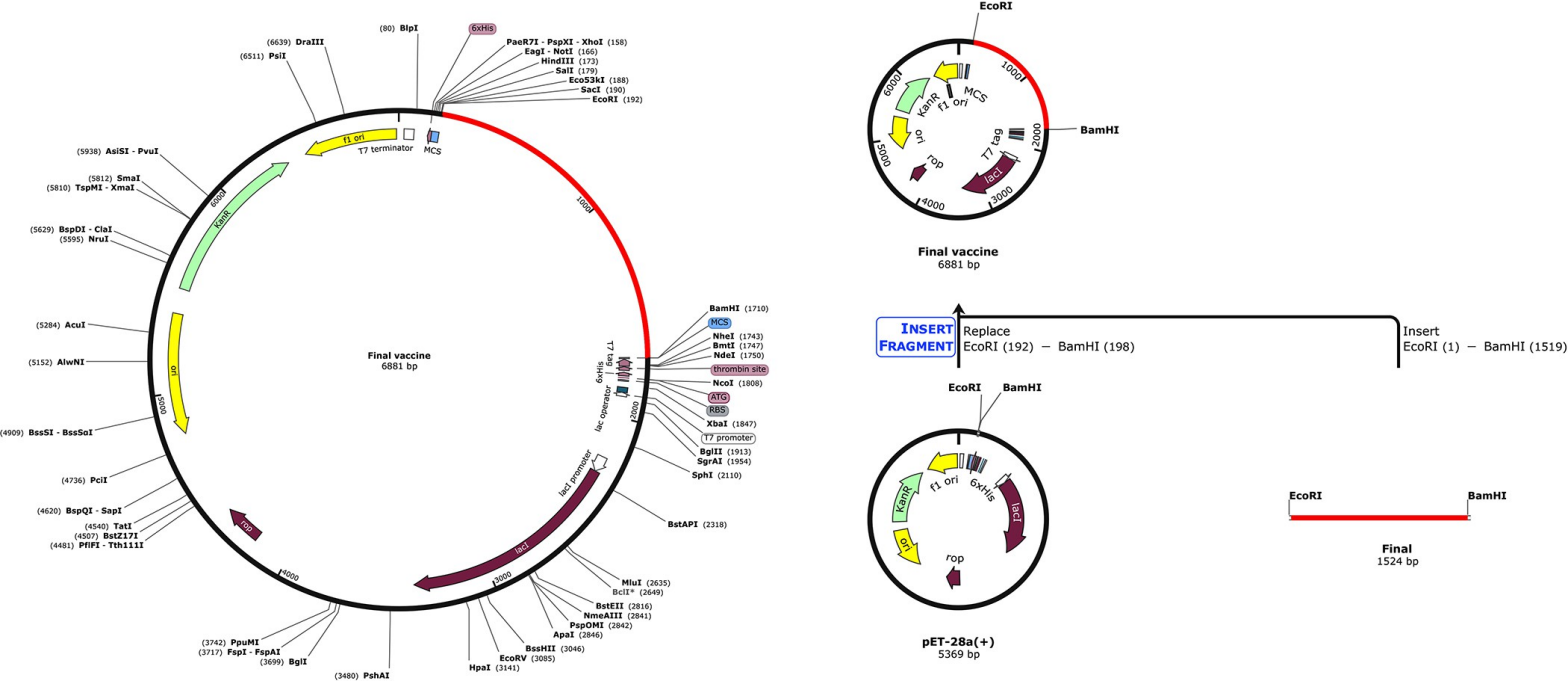
In silico cloning of the final vaccine construct into pET28a (+) expression vector.

Based on the Jcat server, GC content of 52.44% and CAI value of 0.93 were reported, indicating the stability of the designed vaccine in *E*. *coli* expression system. Subsequently, *EcoRI* and *BamHI* restriction sites were added to the N and C terminals of the final codon sequence of the vaccine and SnapGene software was used to integrate the adapted DNA sequence into the pET-28a (+) vector (Fig 10).

### MRNA prediction of the designed vaccine

**Fig 11:**
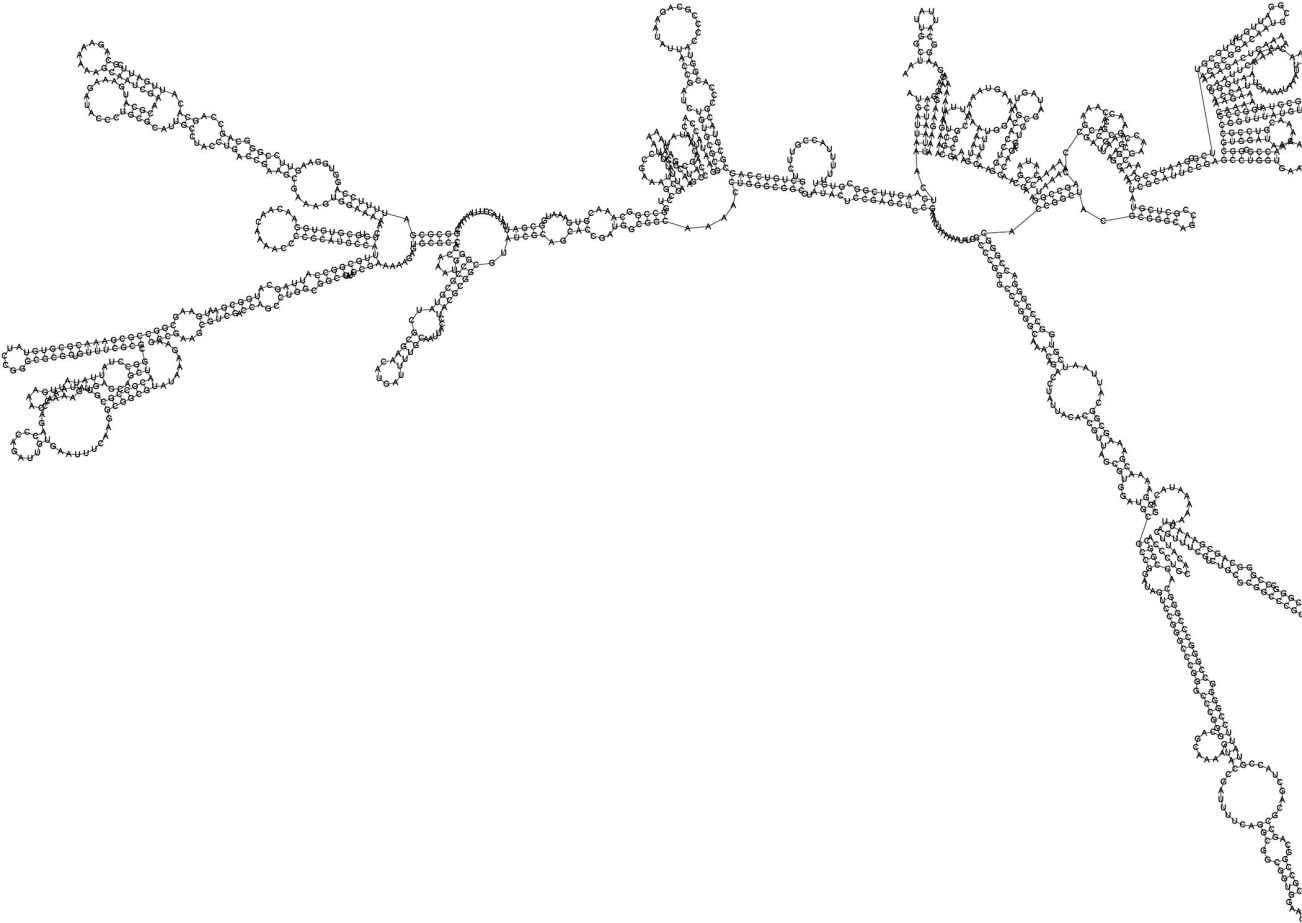
Secondary structure of vaccine mRNA.

According to RNAfold and Mfold v2.3 servers, the best vaccine mRNA secondary structure had the minimum free energy (MFE) of -522.93 kcal/mol and -523.60 kcal/mol, respectively (Fig 11). Based on studies, lower MFE indicates higher thermodynamic stability of mRNA [25, 49, 62]. Therefore, according to the findings, it can be concluded that our predicted vaccine can be very stable after transcription in the body.

## Discussion

Among the pathogenic factors of *G*. *vaginalis*, sialidase and VLY play an important role in the infection of BV [15]. In general, sialidase and vaginolysin cause the breakdown of the protective mucous layer of the vaginal epithelium and the lysis of target cells such as vaginal epithelial cells [63, 64]. Despite the existence of metronidazole and clindamycin, the first-line treatment for *G*. *vaginalis*, the recurrence rate of >50% is still an important challenge for this issue [65]. Therefore, vaccine production against sialidase and VLY can play an important role in its prevention and treatment.

The use of immunoinformatics methods in the development of multi-epitope vaccines has led to the reduction of adverse reactions (eg. allergy) and antigenic load. These types of vaccines are made of different short peptide fragments that cause humoral and cellular immune responses [66]. However, various studies have shown the successful efficacy of multi-epitope vaccine against various pathogens [67–72].

Based on the mentioned servers, 21 epitopes (six cell B epitopes, eight MHC I epitopes and seven MHC II epitopes) were considered. All selected epitopes were antigenic, non-allergenic and non-toxic. The multi-epitope vaccine was designed by several appropriate linkers such as AAY, GPGPG and KK and cholera enterotoxin B subunit adjuvant, which play an important role in increasing immunogenicity. Based on bioinformatic tools, the molecular weight of the candidate vaccine was reported to be 52.7 kDa, which compared to the ideal conditions (less than 110 kDa) [25], is considered as a suitable and efficient vaccine. The theoretical pI of the vaccine was determined to be 9.28, which indicates its alkaline nature. The AI index was 57.58 and the GRAVY index was -0.726, which indicates the thermally stable nature and effective interaction of the vaccine with water. On the other hand, the findings showed that the half-life of the candidate vaccine in mammalian reticulocytes, yeast and *E*. *coli* is such that the vaccine can be exposed to the immune system for a long time. Also, the instability index of 35 was reported, which indicates the stability of the vaccine under standard conditions (less than 40).

According to the PSIPRED server, the secondary structure of the designed vaccine consisted of 33.26% α-helix, 14.46% β-strand, 52.28% random coil. After determining the 3D structure and its refinement, quality optimization and validation were evaluated. Ramachandran diagram created by the PROCHECK server showed that 87.0%, 10.4%, 1.2% and 1.4% of protein residues were located in the most favored region, additional allowed, generously and disallowed (outlier) area of the final vaccine, respectively. The score of ERRAT, Verify-3D and Z-score was 95.335, 84.26% and -7.53, respectively, which indicates the good quality of the designed 3D model.

Based on studies to date, 10 TLRs (TLR1-TLR10) have been identified in humans [73]. Notably, TLR4, a key member of the innate immune response, was docked with the designed multi-epitope vaccine. The results of molecular docking between TLR4 and vaccine indicate a high degree of binding affinity and stability of the complex. In addition, the results of MD simulations confirm that the as designed vaccine maintains its functional state in solution.

The results obtained from the C-IMMSIM server showed an increase in the memory of B cells and T cells, as well as a higher antibody level in secondary and tertiary immune responses compared to the primary immune response. Also, a significant increase in the level of IFN-γ and IL-2 was shown after the third injection of the vaccine. Overall, these results indicate that the candidate multi-epitope vaccine can create an effective immune response against BV caused by *G*. *vaginalis*.

The proposed vaccine was cloned into pET-281(+) overexpression plasmid to optimize the vaccine peptide codon. The CAI and GC values of the contents indicate that the designed vaccine can be well expressed in *E*. *coil* K12 strain. Finally, the lowest free energy score of the vaccine mRNA secondary structure indicated that the vaccine is expected to behave stably in the body.

## Conclusion

Overall, the results of this study provided important insight into the design of an effective and potential multi-epitope vaccine against BV caused by *G*. *vaginalis*. This study showed that the candidate vaccine is structurally stable and has the potential to stimulate long-term immunity in the host, but wet laboratory validation is needed to justify this.

## Limitations

Although the servers used in this study are highly accurate, they have limitations that cannot be compared with the experimental method. For example, the C-IMMSIM server simulator is limited because it lacks the disease layer and is unable to detect vaccine efficacy. Other limitations include the NMA method, which is probably the least computationally expensive method for studying the dynamics of macromolecules, but the MD method is more accurate than NMA, so that it can cover a significantly larger volume of structural space.

The major limitation of the current study is the lack of confirmation and experimental evaluation of the safety and efficacy of the designed multi-epitope vaccine. Therefore, major steps such as laboratory and animal studies are needed to justify our findings to determine safety, efficacy and immunogenicity as a preventive measure. Overall, the application of these results is pending validation in the wet lab experimental models.

## References

[1] Ness RB, Kip KE, Hillier SL, Soper DE, Stamm CA, Sweet RL, et al. A cluster analysis of bacterial vaginosis–associated microflora and pelvic inflammatory disease. American journal of epidemiology. 2005;162(6):585–90. doi: 10.1093/aje/kwi243 16093289

[2] Ma B, Forney LJ, Ravel J. Vaginal microbiome: rethinking health and disease. Annual review of microbiology. 2012;66(1):371–89. doi: 10.1146/annurev-micro-092611-150157 22746335 PMC3780402

[3] Ravel J, Moreno I, Simón C. Bacterial vaginosis and its association with infertility, endometritis, and pelvic inflammatory disease. American journal of obstetrics and gynecology. 2021;224(3):251–7. doi: 10.1016/j.ajog.2020.10.019 33091407

[4] Celeste C, Ming D, Broce J, Ojo DP, Drobina E, Louis-Jacques AF, et al. Ethnic disparity in diagnosing asymptomatic bacterial vaginosis using machine learning. NPJ Digital Medicine. 2023;6(1):211. doi: 10.1038/s41746-023-00953-1 37978250 PMC10656445

[5] Peebles K, Velloza J, Balkus JE, McClelland RS, Barnabas RV. High global burden and costs of bacterial vaginosis: a systematic review and meta-analysis. Sexually transmitted diseases. 2019;46(5):304–11. 10.1097/OLQ.0000000000000972 30624309

[6] Schwiertz A, Taras D, Rusch K, Rusch V. Throwing the dice for the diagnosis of vaginal complaints? Annals of clinical microbiology and antimicrobials. 2006;5:1–7. 16503990 10.1186/1476-0711-5-4 PMC1395331

[7] Usyk M, Schlecht N, Pickering S, Williams L, Sollecito C, Gradissimo A. molBV reveals immune landscape of bacterial vaginosis and predicts human papillomavirus infection natural history. Nat Commun. 2022; 13: 233. doi: 10.1038/s41467-021-27628-3 35017496 PMC8752746

[8] Yeoman CJ, Yildirim S, Thomas SM, Durkin AS, Torralba M, Sutton G, et al. Comparative genomics of Gardnerella vaginalis strains reveals substantial differences in metabolic and virulence potential. PloS one. 2010;5(8):e12411. doi: 10.1371/journal.pone.0012411 20865041 PMC2928729

[9] Leopold S. Heretofore undescribed organism isolated from the genitourinary system. 1953. 13015741

[10] Shvartsman E, Hill JE, Sandstrom P, MacDonald KS. Gardnerella revisited: species heterogeneity, virulence factors, mucosal immune responses, and contributions to bacterial vaginosis. Infection and Immunity. 2023;91(5):e00390–22. doi: 10.1128/iai.00390-22 37071014 PMC10187134

[11] Pleckaityte M. Cholesterol-dependent cytolysins produced by vaginal bacteria: certainties and controversies. Frontiers in cellular and infection microbiology. 2020;9:452. doi: 10.3389/fcimb.2019.00452 31998661 PMC6966277

[12] Shishpal P, Kasarpalkar N, Singh D, Bhor VM. Characterization of Gardnerella vaginalis membrane vesicles reveals a role in inducing cytotoxicity in vaginal epithelial cells. Anaerobe. 2020;61:102090. doi: 10.1016/j.anaerobe.2019.102090 31442559

[13] Shishpal P, Patel V, Singh D, Bhor VM. pH Stress mediated alteration in protein composition and reduction in cytotoxic potential of Gardnerella vaginalis membrane vesicles. Frontiers in Microbiology. 2021;12:723909. doi: 10.3389/fmicb.2021.723909 34795647 PMC8593039

[14] Amabebe E, Anumba DO. The vaginal microenvironment: the physiologic role of lactobacilli. Frontiers in medicine. 2018;5:181. doi: 10.3389/fmed.2018.00181 29951482 PMC6008313

[15] Ma X, Wang X, Ye S, Liu J, Yuan H, Wang N. Biofilm and pathogenic factor analysis of Gardnerella vaginalis associated with bacterial vaginosis in Northeast China. Frontiers in Microbiology. 2022;13:1033040. doi: 10.3389/fmicb.2022.1033040 36619994 PMC9815022

[16] Chen L, Li J, Xiao B. The role of sialidases in the pathogenesis of bacterial vaginosis and their use as a promising pharmacological target in bacterial vaginosis. Frontiers in Cellular and Infection Microbiology. 2024;14:1367233. doi: 10.3389/fcimb.2024.1367233 38495652 PMC10940449

[17] Robinson LS, Schwebke J, Lewis WG, Lewis AL. Identification and characterization of NanH2 and NanH3, enzymes responsible for sialidase activity in the vaginal bacterium Gardnerella vaginalis. Journal of Biological Chemistry. 2019;294(14):5230–45. doi: 10.1074/jbc.RA118.006221 30723162 PMC6462536

[18] Guo N, Niu Z, Yan Z, Liu W, Shi L, Li C, et al. Immunoinformatics Design and In Vivo Immunogenicity Evaluation of a Conserved CTL Multi-Epitope Vaccine Targeting HPV16 E5, E6, and E7 Proteins. Vaccines. 2024;12(4):392. doi: 10.3390/vaccines12040392 38675774 PMC11053576

[19] Oyarzún P, Kobe B. Recombinant and epitope-based vaccines on the road to the market and implications for vaccine design and production. Human vaccines & immunotherapeutics. 2016;12(3):763–7. doi: 10.1080/21645515.2015.1094595 26430814 PMC4964635

[20] De Groot AS, Moise L, McMurry JA, Martin W. Epitope-based immunome-derived vaccines: a strategy for improved design and safety. Clinical applications of immunomics. 2009:39–69.

[21] Rts S. Efficacy and safety of RTS, S/AS01 malaria vaccine with or without a booster dose in infants and children in Africa: final results of a phase 3, individually randomised, controlled trial. Lancet. 2015;386(9988):31–45. doi: 10.1016/S0140-6736(15)60721-8 25913272 PMC5626001

[22] Kran A-MB, Sørensen B, Nyhus J, Sommerfelt MA, Baksaas I, Bruun JN, et al. HLA-and dose-dependent immunogenicity of a peptide-based HIV-1 immunotherapy candidate (Vacc-4x). Aids. 2004;18(14):1875–83. doi: 10.1097/00002030-200409240-00003 15353973

[23] Nardin EH, Oliveira GA, Calvo-Calle JM, Castro ZR, Nussenzweig RS, Schmeckpeper B, et al. Synthetic malaria peptide vaccine elicits high levels of antibodies in vaccinees of defined HLA genotypes. The Journal of infectious diseases. 2000;182(5):1486–96. doi: 10.1086/315871 11023472

[24] Saha S, Raghava GPS. Prediction of continuous B‐cell epitopes in an antigen using recurrent neural network. Proteins: Structure, Function, and Bioinformatics. 2006;65(1):40–8. 10.1002/prot.21078 16894596

[25] Motamedi H, Ari MM, Shahlaei M, Moradi S, Farhadikia P, Alvandi A, et al. Designing multi-epitope vaccine against important colorectal cancer (CRC) associated pathogens based on immunoinformatics approach. BMC bioinformatics. 2023;24(1):65. doi: 10.1186/s12859-023-05197-0 36829112 PMC9951438

[26] Nemati Zargaran F, Akya A, Ghadiri K, Ranjbarian P, Rostamian M. Detecting the dominant T and B epitopes of Klebsiella pneumoniae ferric enterobactin protein (FepA) and introducing a single epitopic peptide as vaccine candidate. International Journal of Peptide Research and Therapeutics. 2021;27(4):2209–21. doi: 10.1007/s10989-021-10247-3 34226823 PMC8243051

[27] EL‐Manzalawy Y, Dobbs D, Honavar V. Predicting linear B‐cell epitopes using string kernels. Journal of Molecular Recognition: An Interdisciplinary Journal. 2008;21(4):243–55. 10.1002/jmr.893 PMC2683948 18496882

[28] Singh H, Ansari HR, Raghava GP. Improved method for linear B-cell epitope prediction using antigen’s primary sequence. PloS one. 2013;8(5):e62216. doi: 10.1371/journal.pone.0062216 23667458 PMC3646881

[29] Fleri W, Paul S, Dhanda SK, Mahajan S, Xu X, Peters B, et al. The immune epitope database and analysis resource in epitope discovery and synthetic vaccine design. Frontiers in immunology. 2017;8:278. doi: 10.3389/fimmu.2017.00278 28352270 PMC5348633

[30] Tan C, Liu T, Chen S, Zhou J, Zhang S, Hu Y, et al. Development of multi-epitope mRNA vaccine against Clostridioides difficile using reverse vaccinology and immunoinformatics approaches. Synthetic and Systems Biotechnology. 2024;9(4):667–83. doi: 10.1016/j.synbio.2024.05.008 38817826 PMC11137598

[31] Yano A, Onozuka A, Asahi-Ozaki Y, Imai S, Hanada N, Miwa Y, et al. An ingenious design for peptide vaccines. Vaccine. 2005;23(17–18):2322–6. doi: 10.1016/j.vaccine.2005.01.031 15755620

[32] Fathollahi M, Motamedi H, Hossainpour H, Abiri R, Shahlaei M, Moradi S, et al. Designing a novel multi-epitopes pan-vaccine against SARS-CoV-2 and seasonal influenza: in silico and immunoinformatics approach. Journal of Biomolecular Structure and Dynamics. 2023:1–24. doi: 10.1080/07391102.2023.2258420 37723861

[33] Walker JM. The proteomics protocols handbook: Springer; 2005.

[34] Kyte J, Doolittle RF. A simple method for displaying the hydropathic character of a protein. Journal of molecular biology. 1982;157(1):105–32. doi: 10.1016/0022-2836(82)90515-0 7108955

[35] Jaspard E, Macherel D, Hunault G. Computational and statistical analyses of amino acid usage and physico-chemical properties of the twelve late embryogenesis abundant protein classes. PloS one. 2012;7(5):e36968. doi: 10.1371/journal.pone.0036968 22615859 PMC3353982

[36] Rostamian M, Farasat A, Chegene Lorestani R, Nemati Zargaran F, Ghadiri K, Akya A. Immunoinformatics and molecular dynamics studies to predict T-cell-specific epitopes of four Klebsiella pneumoniae fimbriae antigens. Journal of Biomolecular Structure and Dynamics. 2022;40(1):166–76. doi: 10.1080/07391102.2020.1810126 32820713

[37] Hebditch M, Carballo-Amador MA, Charonis S, Curtis R, Warwicker J. Protein–Sol: a web tool for predicting protein solubility from sequence. Bioinformatics. 2017;33(19):3098–100. doi: 10.1093/bioinformatics/btx345 28575391 PMC5870856

[38] Doytchinova IA, Flower DR. VaxiJen: a server for prediction of protective antigens, tumour antigens and subunit vaccines. BMC bioinformatics. 2007;8:1–7. 17207271 10.1186/1471-2105-8-4 PMC1780059

[39] Magnan CN, Zeller M, Kayala MA, Vigil A, Randall A, Felgner PL, et al. High-throughput prediction of protein antigenicity using protein microarray data. Bioinformatics. 2010;26(23):2936–43. doi: 10.1093/bioinformatics/btq551 20934990 PMC2982151

[40] Gupta S, Kapoor P, Chaudhary K, Gautam A, Kumar R, Consortium OSDD, et al. In silico approach for predicting toxicity of peptides and proteins. PloS one. 2013;8(9):e73957. doi: 10.1371/journal.pone.0073957 24058508 PMC3772798

[41] Bui H-H, Sidney J, Dinh K, Southwood S, Newman MJ, Sette A. Predicting population coverage of T-cell epitope-based diagnostics and vaccines. BMC bioinformatics. 2006;7:1–5. 16545123 10.1186/1471-2105-7-153 PMC1513259

[42] Ma Y, Liu Y, Cheng J. Protein secondary structure prediction based on data partition and semi-random subspace method. Scientific reports. 2018;8(1):9856. doi: 10.1038/s41598-018-28084-8 29959372 PMC6026213

[43] Buchan DW, Jones DT. The PSIPRED protein analysis workbench: 20 years on. Nucleic acids research. 2019;47(W1):W402–W7. doi: 10.1093/nar/gkz297 31251384 PMC6602445

[44] Kim DE, Chivian D, Baker D. Protein structure prediction and analysis using the Robetta server. Nucleic acids research. 2004;32(suppl_2):W526–W31. doi: 10.1093/nar/gkh468 15215442 PMC441606

[45] Heo L, Park H, Seok C. GalaxyRefine: Protein structure refinement driven by side-chain repacking. Nucleic acids research. 2013;41(W1):W384–W8. doi: 10.1093/nar/gkt458 23737448 PMC3692086

[46] Davis IW, Leaver-Fay A, Chen VB, Block JN, Kapral GJ, Wang X, et al. MolProbity: all-atom contacts and structure validation for proteins and nucleic acids. Nucleic acids research. 2007;35(suppl_2):W375-W83. 17452350 10.1093/nar/gkm216 PMC1933162

[47] Velázquez-Libera JL, Durán-Verdugo F, Valdés-Jiménez A, Núñez-Vivanco G, Caballero J. LigRMSD: A web server for automatic structure matching and RMSD calculations among identical and similar compounds in protein-ligand docking. Bioinformatics. 2020;36(9):2912–4. doi: 10.1093/bioinformatics/btaa018 31926012

[48] Kufareva I, Abagyan R. Methods of protein structure comparison. Homology modeling: Methods and protocols. 2012:231–57. doi: 10.1007/978-1-61779-588-6_10 22323224 PMC4321859

[49] Motamedi H, Alvandi A, Fathollahi M, Ari MM, Moradi S, Moradi J, et al. In silico designing and immunoinformatics analysis of a novel peptide vaccine against metallo-beta-lactamase (VIM and IMP) variants. PLoS One. 2023;18(7):e0275237. doi: 10.1371/journal.pone.0275237 37471423 PMC10358925

[50] Colovos C, Yeates TO. Verification of protein structures: patterns of nonbonded atomic interactions. Protein science. 1993;2(9):1511–9. doi: 10.1002/pro.5560020916 8401235 PMC2142462

[51] Wiederstein M, Sippl MJ. ProSA-web: interactive web service for the recognition of errors in three-dimensional structures of proteins. Nucleic acids research. 2007;35(suppl_2):W407–W10. doi: 10.1093/nar/gkm290 17517781 PMC1933241

[52] Eisenberg D, Lüthy R, Bowie JU. [20] VERIFY3D: assessment of protein models with three-dimensional profiles. Methods in enzymology. 277: Elsevier; 1997. p. 396–404. 9379925 10.1016/s0076-6879(97)77022-8

[53] Fathollahi M, Fathollahi A, Motamedi H, Moradi J, Alvandi A, Abiri R. In silico vaccine design and epitope mapping of New Delhi metallo-beta-lactamase (NDM): an immunoinformatics approach. BMC bioinformatics. 2021;22:1–24. 34563132 10.1186/s12859-021-04378-z PMC8465709

[54] Pinzi L, Rastelli G. Molecular docking: shifting paradigms in drug discovery. International journal of molecular sciences. 2019;20(18):4331. doi: 10.3390/ijms20184331 31487867 PMC6769923

[55] Abraham MJ, Murtola T, Schulz R, Páll S, Smith JC, Hess B, et al. GROMACS: High performance molecular simulations through multi-level parallelism from laptops to supercomputers. SoftwareX. 2015;1:19–25.

[56] López-Blanco JR, Aliaga JI, Quintana-Ortí ES, Chacón P. iMODS: internal coordinates normal mode analysis server. Nucleic acids research. 2014;42(W1):W271–W6. doi: 10.1093/nar/gku339 24771341 PMC4086069

[57] Rapin N, Lund O, Bernaschi M, Castiglione F. Computational immunology meets bioinformatics: the use of prediction tools for molecular binding in the simulation of the immune system. PloS one. 2010;5(4):e9862. doi: 10.1371/journal.pone.0009862 20419125 PMC2855701

[58] Grote A, Hiller K, Scheer M, Münch R, Nörtemann B, Hempel DC, et al. JCat: a novel tool to adapt codon usage of a target gene to its potential expression host. Nucleic acids research. 2005;33(suppl_2):W526–W31. doi: 10.1093/nar/gki376 15980527 PMC1160137

[59] Gote V, Bolla PK, Kommineni N, Butreddy A, Nukala PK, Palakurthi SS, et al. A comprehensive review of mRNA vaccines. International journal of molecular sciences. 2023;24(3):2700. doi: 10.3390/ijms24032700 36769023 PMC9917162

[60] Lorenz R, Bernhart SH, Höner zu Siederdissen C, Tafer H, Flamm C, Stadler PF, et al. ViennaRNA Package 2.0. Algorithms for molecular biology. 2011;6:1–14. 22115189 10.1186/1748-7188-6-26 PMC3319429

[61] Agu P, Afiukwa C, Orji O, Ezeh E, Ofoke I, Ogbu C, et al. Molecular docking as a tool for the discovery of molecular targets of nutraceuticals in diseases management. Scientific Reports. 2023;13(1):13398. doi: 10.1038/s41598-023-40160-2 37592012 PMC10435576

[62] Zhu F, Tan C, Li C, Ma S, Wen H, Yang H, et al. Design of a multi-epitope vaccine against six Nocardia species based on reverse vaccinology combined with immunoinformatics. Frontiers in Immunology. 2023;14:1100188. doi: 10.3389/fimmu.2023.1100188 36845087 PMC9952739

[63] Schellenberg JJ, Paramel Jayaprakash T, Withana Gamage N, Patterson MH, Vaneechoutte M, Hill JE. Gardnerella vaginalis subgroups defined by cpn 60 sequencing and sialidase activity in isolates from Canada, Belgium and Kenya. PloS one. 2016;11(1):e0146510. doi: 10.1371/journal.pone.0146510 26751374 PMC4709144

[64] Gelber SE, Aguilar JL, Lewis KL, Ratner AJ. Functional and phylogenetic characterization of Vaginolysin, the human-specific cytolysin from Gardnerella vaginalis. Journal of bacteriology. 2008;190(11):3896–903. doi: 10.1128/JB.01965-07 18390664 PMC2395025

[65] Machado D, Castro J. Palmeira-de-Oliveira AA Bacterial vaginosis biofilms: Challenges to current therapies and emerging solutions. Front Microbiol. 2016;6:1528. 26834706 10.3389/fmicb.2015.01528 PMC4718981

[66] Oli AN, Obialor WO, Ifeanyichukwu MO, Odimegwu DC, Okoyeh JN, Emechebe GO, et al. Immunoinformatics and vaccine development: an overview. ImmunoTargets and therapy. 2020:13–30. doi: 10.2147/ITT.S241064 32161726 PMC7049754

[67] Jiang P, Cai Y, Chen J, Ye X, Mao S, Zhu S, et al. Evaluation of tandem Chlamydia trachomatis MOMP multi-epitopes vaccine in BALB/c mice model. Vaccine. 2017;35(23):3096–103. doi: 10.1016/j.vaccine.2017.04.031 28456528

[68] Lennerz V, Gross S, Gallerani E, Sessa C, Mach N, Boehm S, et al. Immunologic response to the survivin-derived multi-epitope vaccine EMD640744 in patients with advanced solid tumors. Cancer immunology, immunotherapy. 2014;63:381–94. doi: 10.1007/s00262-013-1516-5 24487961 PMC11029529

[69] Slingluff CL Jr, Lee S, Zhao F, Chianese-Bullock KA, Olson WC, Butterfield LH, et al. A randomized phase II trial of multiepitope vaccination with melanoma peptides for cytotoxic T cells and helper T cells for patients with metastatic melanoma (E1602). Clinical Cancer Research. 2013;19(15):4228–38. doi: 10.1158/1078-0432.CCR-13-0002 23653149 PMC3813832

[70] Toledo H, Baly A, Castro O, Resik S, Laferté J, Rolo F, et al. A phase I clinical trial of a multi-epitope polypeptide TAB9 combined with Montanide ISA 720 adjuvant in non-HIV-1 infected human volunteers. Vaccine. 2001;19(30):4328–36. doi: 10.1016/s0264-410x(01)00111-6 11457560

[71] Dasari V, McNeil LK, Beckett K, Solomon M, Ambalathingal G, Thuy TL, et al. Lymph node targeted multi-epitope subunit vaccine promotes effective immunity to EBV in HLA-expressing mice. Nature Communications. 2023;14(1):4371. doi: 10.1038/s41467-023-39770-1 37553346 PMC10409721

[72] Nie J, Wang Q, Jin S, Yao X, Xu L, Chang Y, et al. Self-assembled multiepitope nanovaccine based on NoV P particles induces effective and lasting protection against H3N2 influenza virus. Nano research. 2023;16(5):7337–46. doi: 10.1007/s12274-023-5395-6 36820263 PMC9933037

[73] Duan T, Du Y, Xing C, Wang HY, Wang R-F. Toll-like receptor signaling and its role in cell-mediated immunity. Frontiers in Immunology. 2022;13:812774. doi: 10.3389/fimmu.2022.812774 35309296 PMC8927970

