# Correction: Immunoinformatic evaluation for the development of a potent multi-epitope vaccine against bacterial vaginosis caused by *Gardnerella vaginalis*

**DOI:** 10.1371/journal.pone.0331745

**Published:** 2025-09-04

**Authors:** Hamid Motamedi, Saeed Shoja, Maryam Abbasi

There are errors in the author affiliations. The correct affiliations are as follows: Hamid Motamedi^1^, Saeed Shoja^2^, Maryam Abbasi^3^

**1** Asadabad School of Medical Sciences, Asadabad, Iran, **2** Infectious and Tropical Diseases Research Center, Hormozgan Health Institute, Hormozgan University of Medical Sciences, Bandar Abbas, Iran, **3** Endocrinology and Metabolism Research Center, Hormozgan University of Medical Sciences, Bandar Abbas, Iran
